# Internationally studied parameters related to the COVID-19 pandemic in nursing homes: a scoping review

**DOI:** 10.1186/s13643-026-03171-4

**Published:** 2026-03-27

**Authors:** Almuth Berg, Christin Richter, Gabriele Meyer

**Affiliations:** Institute of Health, Midwifery and Nursing Science, Medical Faculty, Martin Luther University Halle-Wittenberg, Halle (Saale), Germany

**Keywords:** COVID-19, Coronavirus, Pandemics, Nursing homes, Long-term care, Scoping review

## Abstract

**Background:**

Nursing homes were severely affected by the COVID-19 pandemic. Standardised parameters are essential to understand and monitor the unintended consequences of pandemic control measures and changes in work processes. In this scoping review, we aimed to identify COVID-19-related parameters studied in nursing homes that could form a minimum data set suitable for database development. We focused on the perspectives of all interest-holders: facilities, residents, their relatives and nursing home staff.

**Methods:**

We searched MEDLINE and CINAHL and included quantitative studies published in English since the beginning of the pandemic (2020 to 2024). The extracted parameters were initially categorised according to five dimensions: pandemic-related data, facility level, staff level, residents and relatives. Within each dimension, the original terms were compared and inductively organised into (sub-)categories based on conceptual similarities, with synonymous terms subsequently standardised.

**Results:**

From 82 included articles, 96 different parameters related to COVID-19 in nursing homes were identified. Infection and mortality rates emerged as the pandemic-related data most often reported, particularly within this dimension but also across all dimensions. However, we found a broad range of resident-related parameters. Our most often identified facility-related parameters include the number of staff and the provision of personal protective equipment. Staff-related parameters most often studied were personal burden and stress. Only a few parameters (*n* = 9) were considered for relatives.

**Conclusions:**

The diversity of the reported parameters indicates that a comprehensive database is required to adequately assess a pandemic situation in this vulnerable population. In terms of pandemic preparedness, our overview of the reported parameters offers a basis for the development of country- and context-specific data capture approaches.

**Supplementary Information:**

The online version contains supplementary material available at 10.1186/s13643-026-03171-4.

## Background

Around the world, nursing homes were severely affected by the coronavirus disease 2019 (COVID-19) pandemic [1]. Nursing homes are defined as long-term care facilities that provide 24/7 support by healthcare professionals, mainly on-site registered nurses, for people who need constant assistance with activities of daily living due to old age or chronic medical conditions [2]. Residents of these facilities represent a particularly vulnerable population. They were at higher risk of COVID-19 outbreaks, development of severe courses of the disease and mortality associated with a COVID-19 infection. A systematic review [3] from the first wave of the pandemic, with 49 observational studies in aged care facilities across 14 countries and four continents, revealed that 45% (95% CI 32–58%) of residents per facility became infected and 23% (95% CI 18–28%) of those infected died. Nearly one third of the cases (31% (95% CI 28–34%)) were asymptomatic and 37% (95% CI 35–39%) led to hospitalisation. Additionally, the pandemic-related prevention and control measures had a significant impact on residents’ mental and physical health and well-being, and posed challenges for staff [4]. Loneliness, sadness and depressive symptoms, but also fear, were often reported as reactions of residents to the contact and visiting restrictions [5]. Residents with cognitive impairments may have suffered more, but there were also contradictory reports. Nursing home staff described fear of infecting themselves, the residents, or their own families. Furthermore, staff reported a considerable increase in workload [5].

In particular, the lockdown of nursing homes raises questions about the benefit–harm ratio of pandemic control measures. Based on the US National Institutes of Health guidelines [6], Zhang et al. [7] selected four *critical outcomes* related to COVID-19: infection rate, hospitalisation rate, case-fatality rate and mortality rate. These critical outcomes have been the focus of the majority of COVID-19-related publications, including both regular surveillance reports [see, among others [8]] and numerous research papers, as well as reviews [see, among others [9]]. However, reporting on the unintended negative consequences of the pandemic control measures and the impact of the changed work processes on nursing home residents and staff is important as well [5, 10]. To enhance better navigation in future pandemics, consistent collection of outcomes related to this type of information is essential—yet no consensus exists on a pandemic-related minimum data set (MDS). An MDS describes a standardised, comprehensive tool for an adult’s assessment, care planning and evaluation, commonly used in long-term care. Defining a core set of pandemic-related parameters is therefore needed. In terms of pandemic preparedness, a corresponding overview of potentially relevant parameters could provide a basis for the development of country- and context-specific approaches for standardised data capture in nursing homes during crises. To our knowledge, there is currently no overview synthesising critical outcomes alongside outcomes related to further effects experienced by residents, relatives and nursing home staff over the entire pandemic period.

Our objective was to systematically identify and map all pandemic-related parameters that are considered relevant at the international level for nursing homes. We therefore explored which parameters (such as variables and outcomes) have been studied in quantitative studies on nursing homes during the COVID-19 pandemic.

## Methods

A scoping review was chosen, since it can identify knowledge gaps, scope a body of literature, clarify concepts and investigate research conduct. The scoping review was carried out in accordance with the methodological recommendations of the Joanna Briggs Institute [11]. In particular, the framework provides detailed guidance on the following components: inclusion criteria, evidence search, selection, data extraction, analysis and summary of results. The PRISMA-ScR statement guided our reporting [12].

### Eligibility criteria

We included quantitative primary studies and quantitative data of mixed method studies published in English since the beginning of the pandemic. Studies reporting on COVID-19-related parameters in nursing homes in relation to facilities, staff, residents, or their relatives were included.

We did not aim to analyse the reporting of critical outcomes and COVID-19-related risk factors used to monitor infection dynamics per se, but to comprehensively identify relevant parameters for assessing the pandemic-related situation in nursing homes. As critical outcomes and risk factors are commonly reported, inclusion was restricted to studies providing a broader depiction of the nursing home situation, namely those that either (a) reported critical outcomes and risk factors alongside additional relevant parameters or (b) reported only other relevant parameters. Consequently, we excluded studies reporting exclusively on analyses of COVID-19-related risk factors, infection and mortality rates, or pandemic progression and its determinants. Studies describing the effectiveness of COVID-19-related interventions, protection measures, vaccination and serological testing were also excluded.

### Information sources, search strategy and selection of sources of evidence

We searched MEDLINE via PubMed (last search: 14 February 2024) and CINAHL via EBSCO (last search: 27 March 2024) using a sensitive search strategy containing the respective medical subject headings for the two components: *COVID-19* and *nursing home* (Table 1). No filters or limits were applied. Furthermore, we searched the reference lists of thematically relevant systematic reviews for additional eligible primary studies.

**Table 1:** Search strategies for MEDLINE and CINAHL

MEDLINE via PubMed	
("COVID-19"[MeSH Terms] OR " SARS-CoV-2"[MeSH Terms] OR "coronavirus"[MeSH Terms]) AND ("nursing homes"[MeSH Terms])	
CINAHL via EBSCO	
1	(MH “COVID-19”)
2	(MH “SARS-CoV-2”)
3	(MH “Coronavirus”)
4	(MH “Nursing Homes”)
5	((MH “Nursing Homes”)) AND (1 OR 2 OR 3)

Two reviewers (AB and CR) independently screened titles, abstracts and full texts. Uncertainty or disagreement was resolved by discussion.

### Data charting process

An extraction sheet was developed and approved by the research team. We extracted basic study characteristics (country, study design, sample size of nursing homes and participants) and all types of parameters (variables and outcomes) reported in the included articles. Any discrepancies were resolved by consensus between the two reviewers (AB and CR).

As we were only interested in the parameters studied and did not report on the study results, no quality appraisal of the included studies was performed.

### Data synthesis

The parameters, extracted verbatim from the original publications (as presented in Additional file 1: Supplementary Table S1), from each included study were initially categorised into five dimensions reflecting the perspectives of the inclusion criteria: pandemic-related data, facility- and staff-level parameters, as well as resident- and relative-related parameters. Within each dimension, the original terms were compared and inductively organised into (sub-)categories based on conceptual similarities, with synonymous terms subsequently standardised. The primary categorisation was performed by two authors (AB and CR), cross-checked by a third author (GM) and finalised collaboratively by all authors.

Study characteristics are reported descriptively. Reporting frequencies are provided for each identified parameter.

## Results

### Study selection

**Fig. 1:**
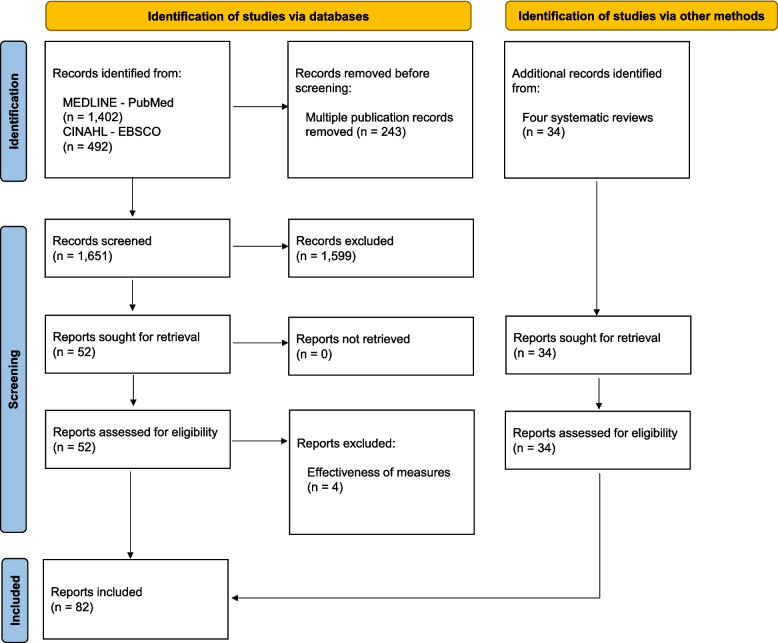
PRISMA flowchart of study selection

Figure 1 summarises the study selection process. The database search resulted in 1651 unique articles. After screening and full-text assessment, 48 articles were included. Additionally, 34 primary studies were identified from the reference lists of four systematic reviews [5, 10, 13, 14], resulting in a total of 82 included articles. (The complete references of the included articles are presented in Additional file 1).

### Study characteristics

**Fig. 2:**
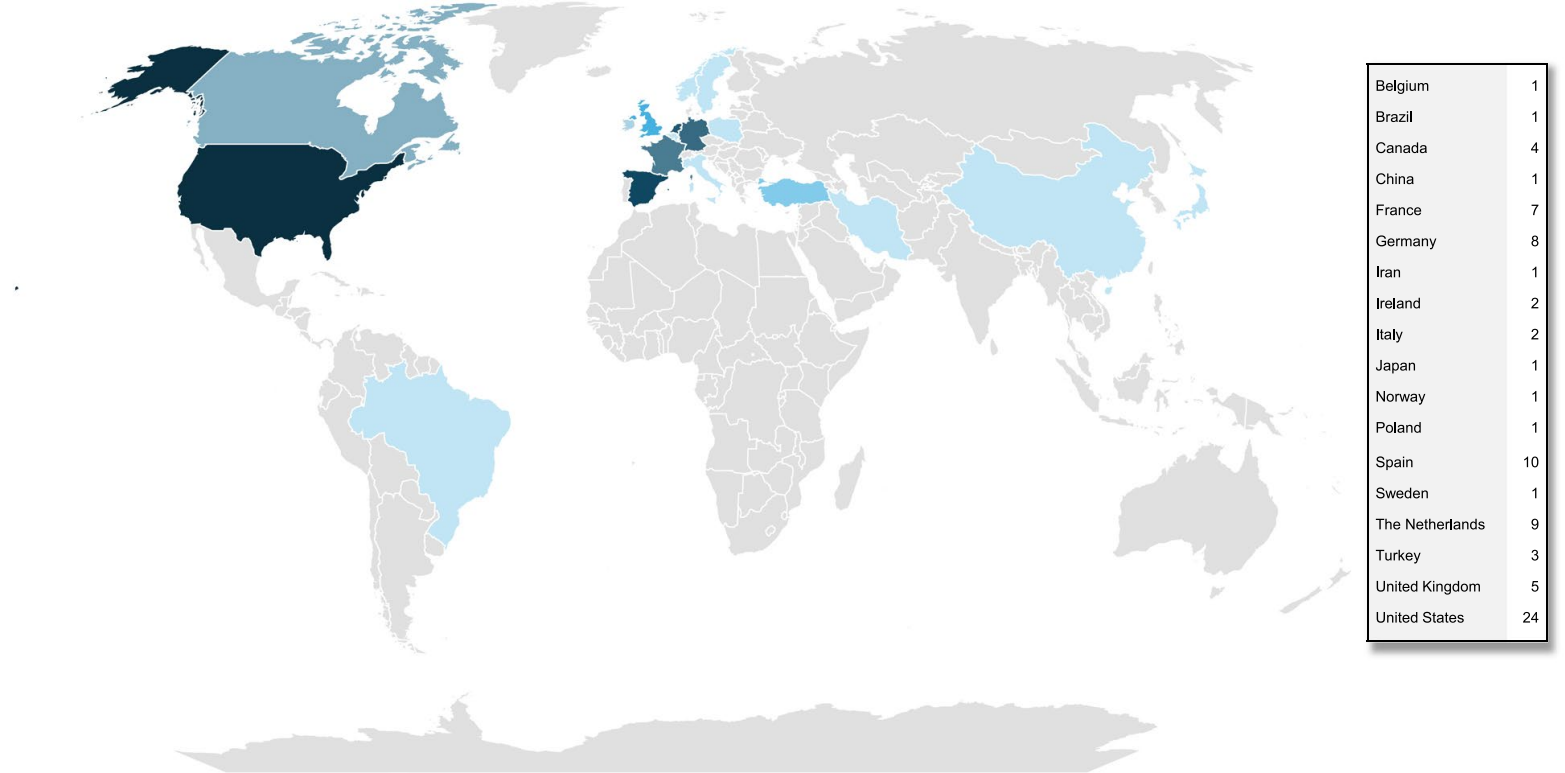
Geographical distribution of the included studies (*n* = 82), 2020 to 2024; darker saturation represents higher values (Source: Own representation using Microsoft PowerPoint)

Supplementary Table S1 (see Additional file 1) provides an overview of the characteristics of the included studies. The studies were conducted in 18 countries (Fig. 2), mostly in Europe (*n* = 47; 57%), followed by the USA (*n* = 24; 29%). Since only four mixed method studies [15–18] were included, the majority were quantitative primary studies, mainly cross-sectional designs (*n* = 37/78; 47%).

The sample size of nursing homes per study, if described, varied from one (*n *= 7 articles) to 15,751 nursing homes [19]. The studies primarily focused on residents (*n* = 54) and/or nursing home staff (*n* = 37). Only a few of the studies addressed relatives [16, 18, 20–27]. In some cases, the managers of the nursing homes were primarily involved [16, 27–31].

### Synthesis of reported parameters related to COVID-19 in nursing homes

The analysis resulted in the identification of *n* = 96 different parameters related to COVID-19 in nursing homes. The parameters are summarised below in the assigned dimension (pandemic-related data, facility level, staff level, resident level, relative level) and presented in the corresponding Tables 2, 3, and 4, indicating the number of underlying references. The reported parameters of each included study are detailed in the analysis matrix in Supplementary Table S1 (see Additional file 1). In addition, Supplementary Table S2 provides an overview of the identified parameters with the corresponding references.

**Table 2:** Identified parameters related to COVID-19 in nursing homes with the number of corresponding references (*n* = 82), 2020 to 2024: pandemic-related data and facility level

Identified parameters		Number of corresponding references
**Pandemic-related data**		
Pandemic progression:		
	Local COVID-19 incidence/prevalence	7
	COVID-19 outbreak/infection rate in nursing home (number of residents)	31
	COVID-19 outbreak/infections among staff/staff absences	16
	Progress in the vaccination campaign for residents and staff	1
Staff level:		
	Symptoms of a COVID-19 infection	4
	Staff fears and concerns (… of infecting themselves, residents, or their relatives)	6
	Impact of vaccination on the daily lives of staff	1
	Impact of the prolonged COVID-19 pandemic and protective restrictions on staff	4
Resident level:		
	Risk of severe COVID-19 progression (especially male gender, age, chronic renal insufficiency, dementia, haematological/oncological diseases)	7
	Symptoms of a COVID-19 infection	18
	Mortality related to COVID-19	16
	Impact of vaccination on the daily lives of residents	2
**Facility level: epidemiological and general data**		
Nursing home characteristics:		
	Nursing home size/number of beds/number of residents	9
	Size of the care units	1
	Ownership of the nursing home	9
	Specialised units	3
	Case mix	3
	Separate sleeping areas/single-occupancy or shared rooms	2
	Nursing home quality of care	2
Staff deployment:		
	Number of staff/staffing ratio/staff shortages	12
	Staffing plan or adjustment of staff assignment (assignment of tasks/changes to the scope of work/changes to processes and procedures/formation of stable teams/availability of external staff/absence rules for staff with COVID-19 infection)	3
Feeling of being well informed and advised		1
Burdens on the management level		2
**Facility level: pandemic-related protective and control measures**		
Use of air filters		1
Contact reduction (e.g. visiting bans/access restrictions for relatives/volunteers/external service providers)		1
Change in contact options (tablet, telephone, digital communication)/offer of alternatives: communication through window panes, outdoor events, hygiene protection wall/separators		2
Cancellation of social activities		1
Cohorting/relocation/transfer/isolation of COVID-19 infected residents within the facility		2
Testing for COVID-19 infections among staff		5
Testing for COVID-19 infections among residents		8
Provision of hygiene material and personal protective equipment		11
Implementation of hygiene rules or information from public authorities/pandemic plan		6
Training of nursing staff (protective clothing, special hygiene instructions, dealing with highly infectious residents)		2
**Facility level: nursing and health care**		
Access restrictions for general practitioners and medical specialists		1
General practitioners’ or medical specialists’ care deficit (in routine or acute cases)		2
Acute inpatient care (hospitalisation rate of residents)		9
End-of-life care/dealing with residents in palliative situations		1
Cooperation with external institutions or persons		3

**Table 3:** Identified parameters related to COVID-19 in nursing homes with the number of corresponding references (*n* = 82), 2020 to 2024: staff level

Identified parameters		Number of corresponding references
**Staff level**		
Psychosocial factors at the workplace:		
	Impact of changing work requirements and flexibility requirements	4
	Work intensity and work intensification	5
	Workload	2
	Psychosocial effects of dealing with palliative care residents	3
	Uncertainties regarding the implementation of frequently changing regulations and guidelines (sometimes daily-changing rules), role ambiguities	4
	Needs of nursing staff (e.g. recognition and appreciation of care, coronavirus bonus, supervision for nursing staff during the pandemic)	5
	Psychosocial effects of interacting with relatives	1
	Considerations of changing or leaving the profession	2
	Job satisfaction	4
	Satisfaction with the quality of nursing care	1
	Effects on the general health status	3
	Burnout	10
	Effects on presenteeism	1
Personal burden, stress, anxiety, depression, fatigue, panic attacks, helplessness, loss of control		14
Physical and mental overload of staff		3
Substance abuse		1
Suicidal thoughts and plans		1
Experiences of prejudice and discrimination		2
Staff well-being		4
Fear for the well-being of the residents		1
Coping strategies, resilience		3

**Table 4:** Identified parameters related to COVID-19 in nursing homes with the number of corresponding references (*n* = 82), 2020 to 2024: resident level and relative level

Identified parameters		Number of corresponding references
**Resident level**		
General health status:		
	Functional status	5
	Nutritional status (body mass index)	8
	Frailty	5
	Cognition and orientation/delirium	10
Participation:		
	Visits (number, location and visitors)	4
	Relationships with significant others and loss of significant others (staff, residents, family, friends)	2
	Possibility of social participation/external participation	3
	Possibility of internal participation	1
	Social isolation, social distancing and loneliness	3
Physical and psychological effects:		
	Mood and enjoyment of life	3
	Sadness, listlessness	2
	Depression/depressive symptoms	11
	Anxiety and stress	6
	COVID-19-related anxiety and coping strategies/resilience	2
	Behaviour and psychiatric symptoms of dementia (BPSD)/psychotropic drugs	5
	Sleep problems	1
	Quality of life and well-being	8
	Need for support	1
	Dealing with protective measures/sense of security/requests	3
	Satisfaction with nursing and health care	1
	Recovery	2
All-cause mortality		4
Evaluation of nursing care:		
	Mobility/falls	3
	Change in the general need for care	2
	Neglect (decubitus, dehydration, urinary tract infection)	3
	Pain	1
Effects on health care provision		1
Dealing with dying and death		3
**Relative level**		
Relatives’ expectations		1
Ways of communication with the facility and residents		2
Perception of the health care situation		3
Support needs of relatives		1
Experience and acceptance of protective measures/sense of security/requests		5
Compliance with local visitation and hygiene protocol and its implications		2
Perceived stressful situation of the relatives (feelings of anger and annoyance/feelings of despair/emotionally stressful conflicts/feelings of helplessness/sadness/frustration/fear/trauma/resilience)		4
Loneliness/social isolation/social distancing		3
Quality of life and well-being of relatives		3

#### Pandemic-related data

The general pandemic-related parameters were grouped in relation to pandemic progression, staff level and resident level (Table 2). Of the included studies, 38% reported the COVID-19 infection rate of nursing home residents. Symptoms of COVID-19 infections and COVID-19 mortality among residents were described in 22% and 20% of the articles, respectively. Likewise, COVID-19 infection rates among staff were reported in 20% of articles.

#### Facility level

The parameters examined in the studies at facility level were further categorised into epidemiological and general data (including nursing home characteristics and staff deployment), pandemic-related protective and control measures, as well as nursing and healthcare (Table 2). The most often reported parameters regarding general facility data were the number of staff (15% of articles) and the size and ownership of the nursing home (11% each). In terms of pandemic-related measures, the provision of hygiene material and personal protective equipment (13% of articles) and the testing of residents for COVID-19 infections (10% of articles) were described most often. With regard to nursing and healthcare, 11% of the studies analysed the hospitalisation rate of residents.

#### Staff level

The most often parameter was personal burden and stress of staff (Table 3), which was addressed in 17% of all included articles and in 38% of the articles exclusively focusing on staff. Burnout was also often considered (12% and 27%, respectively).

#### Resident level

The resident-related parameters can be grouped into general health status, participation, physical and psychological effects and evaluation of nursing care (Table 4). The focus was on depression (20% of resident-related articles; 13% of all articles), cognition and orientation (18%; 12%), quality of life (15%; 10%), and nutritional status (15%; 10%).

#### Relative level

Parameters related to the relatives of nursing home residents were rarely considered, i.e. a few parameters in 1% to 6% of the included articles (Table 4).

## Discussion and implications

Our objective was to obtain a comprehensive overview of internationally studied parameters related to the situation of nursing homes during the COVID-19 pandemic, providing a basis for developing the first public, international MDS of important variables from the perspectives of facilities, residents, their relatives and nursing home staff.

The scope of parameters identified was highly diverse, with critical outcomes such as infection and mortality rates most often reported, even though studies reporting exclusively on analyses of these were already excluded. Nevertheless, the extensive number of different parameters indicates the need for many additional variables in order to adequately assess a pandemic situation and the corresponding measures in this vulnerable population.

The scoping review by Verbiest et al. [10] on the health impact of the first and second wave of COVID-19 among nursing home residents also found that a large number of studies—mainly from Europe and the USA—examined the impact of COVID-19 on infection and mortality rates, symptoms and hospitalisation. Furthermore, a pronounced knowledge gap regarding the perspective of nursing home residents with regard to the psychological and social impact was identified. However, the scoping review exclusively focused on data collected from residents themselves, which may have missed studies collecting proxy data. In this respect, the broad range of resident-related parameters found in our review is likely because we considered both the entire pandemic period and all types of data collection.

In addition to the typical critical outcomes, some World Health Organisation (WHO) documents [1, 32] also address a number of our identified parameters. Prioritising the psychological well-being of people receiving and providing long-term care services is one of the WHO policy objectives for the prevention and management of COVID-19 in long-term care [32]. Key actions in this regard include, among others, ensuring that visiting policies enable a balance between infection prevention and control measures and residents’ needs to maintain their psychological well-being. Stress and burnout among nursing home staff should be monitored, and strategies to provide mental health and psychosocial support to staff delivering long-term care should be implemented. Also, staffing procedures should be reviewed to better manage the burden of care. These action points are also reflected in our staff-related parameters, in particular personal burden and stress of staff.

In the scoping review of challenges and responses of nursing homes during the COVID-19 pandemic by Giri et al. [4], the facility characteristics (e.g. physical space, occupancy, ownership), staffing (e.g. staffing levels, staff-to-resident ratio), as well as external factors (e.g. availability of personal protective equipment), emerged as three of the key themes. Our facility-related parameters most often identified also include the number of staff, number of beds, ownership of the nursing home and the provision of hygiene material and personal protective equipment.

Our focus was on the perspectives of all those affected, but only a few articles could be found on relatives and only a few parameters were considered there. In contrast, the WHO key action points emphasise that family caregivers, who provide psychological and practical support for people living in long-term care facilities, should be enabled to continue such roles through supportive measures [32].

However, our overview of COVID-19-related parameters can serve as a basis to supplement existing protocols for pandemic-related data collections with mainly critical outcomes, e.g. on the severity of breakthrough COVID-19 infections in outbreaks at long-term care facilities [see, among others [33]], with additional variables to be selected. Critical outcomes can be assessed using relatively straightforward procedures, in contrast to determining social and psychological effects on residents and staff [10]. Parameters in this regard would therefore have to be tailored and agreed upon a national basis, possibly similar to the methodological approaches used in the development of an MDS. In order to optimise its accessibility, usability and usefulness, various core principles were formulated as part of the development of an MDS for older adult care home residents in the UK [DACHA study: [34, 35]]. Among other things, the MDS must focus on measuring what matters most for supporting people living in nursing homes. In addition, the MDS must be evidence-based in terms of content and must reduce the data burden and duplication of effort for the nursing home [34]. Consequently, the first work package of the DACHA study consisted of systematic evidence reviews to inform measures and outcomes for inclusion in the MDS. The resulting definition of the MDS content necessitates a collaborative approach to select relevant variables, recognising that interest-holders differ in their priorities and informational needs due to factors such as sensitivities concerning specific variables, commercial implications, regulatory obligations, and oversight responsibilities [34]. On this basis, the MDS will undergo gradual development and piloting in the subsequent work packages, linking routine health service data and data generated by care homes.

In the context of pandemics, longitudinal data on infections and deaths in nursing homes as core data would allow real-time monitoring, early identification of emerging problems and targeted interventions [36]. Moreover, data on the health of residents and staff can be invaluable for developing strategies to protect those at greatest risk and to limit further spread within nursing homes [36]. However, as data collections often prioritise clinical indicators of resident health over social care priorities and what matters to people in nursing homes in terms of quality of life additional parameters reflecting these dimensions should also be incorporated [34].

The articles included in our scoping review are mainly from high-income countries. Therefore, it cannot be ruled out that our overview omits some parameters that are particularly relevant to low- and middle-income countries. While the effects of the pandemic widened disparities in access to services and health outcomes across all health systems, their impact was much greater in systems with limited resources and infrastructure [37]. The lacking capacity to obtain the required vaccination doses and insufficient supply of oxygen tanks in hospitals of low‐ and middle‐income countries raised the likelihood of death among critical COVID‐19 patients [38]. However, several studies show that many of the challenges that appeared to be new and pandemic-related were in fact pre‑existing issues that were highlighted and exacerbated by the pandemic, particularly health inequities. For example, insufficient supply of medical products and workforce to provide care are not new phenomena in low‐ and middle‐income countries, but they were aggravated during the pandemic and further widened the gap between rich and poor countries [37].

Our scoping review used a sensitive search strategy to rigorously identify pandemic-related parameters that have been considered in quantitative studies on nursing homes during the COVID-19 pandemic. We focused on parameters related to the perspectives of the facilities, residents, their relatives and nursing home staff, although certain methodological limitations of this review need to be considered. We included English-language articles only, as we were interested in reports that have been published at an international level. National publications and non-academic material may have covered further parameters, depending on the specific situation in each country. Nevertheless, in order to maintain a high-quality standard, Verbiest et al. [10] also recommend that only empirical and peer-reviewed studies should be included in such analyses.

We restricted our search to two databases and attempted to enhance the comprehensiveness of the parameter mapping by screening the reference lists of thematically relevant systematic reviews for additional primary studies. Inclusion of further databases might have identified additional articles reporting further parameters.

Some publications may have referred to the same data collection. However, our primary objective was to identify the range of pandemic-related parameters that were reported, rather than quantifying them in detail. For this reason, too, we have not differentiated between pandemic waves, but have mapped the entire pandemic period, so that the steady increase in knowledge over the pandemic period is reflected.

Given our focus on quantitative studies, future research might benefit from a scoping review of qualitative studies on the experiences of residents, staff and relatives. Qualitative evidence might generate additional themes that both enrich and expand the parameters identified in this review.

## Conclusions

The parameters studied in international quantitative studies on nursing homes during the COVID-19 pandemic were highly diverse. Although critical outcomes such as infection and mortality rates have been reported most often, the diversity of identified parameters suggests that additional outcomes are needed for a proper assessment of such situations. A robust database is a prerequisite for evidence-informed, ethically responsible and coordinated actions. It allows careful management and description of a pandemic event, as well as the effects of public health, social and other measures. In terms of preparedness for future crises, our overview of the reported parameters offers a basis for supplementing existing surveillance protocols, as well as any existing MDS, with additional pandemic-relevant variables as needed. However, it is imperative that a core set of parameters for nursing homes is defined. The development of country- and context-specific approaches for standardised data capture and agreed-upon selection of appropriate parameters should ensure that data is collected in the most efficient and least onerous manner. Where possible, information gathered routinely by the nursing homes should be used.

## Supplementary Information

Additional file 1: References and characteristics of included studies; overview of identified parameters with corresponding references.

Additional file 2: Preferred Reporting Items for Systematic Reviews and Meta-Analyses extension for Scoping Reviews (PRISMA-ScR) checklist.

## Data Availability

The full dataset used in the review has been provided as an additional file.

## Abbreviations

BPSD: Behaviour and psychiatric symptoms of dementia
COVID-19: Coronavirus disease 2019
MDS: Minimum Data Set
WHO: World Health Organisation
