# Supplementary material for: Internationally studied parameters related to the COVID-19 pandemic in nursing homes: a scoping review
Source: Syst Rev. 2026 Mar 27;15:123. doi: 10.1186/s13643-026-03171-4 (PMC13063767; doi:10.1186/s13643-026-03171-4)
Supplement: Supplementary file 1 — Additional file 1: References and characteristics of included studies; overview of identified parameters with corresponding references. [file 13643_2026_3171_MOESM1_ESM.pdf]

# Additional file 1

## Table of content

Supplementary Table S1. Characteristics and reported parameters of included studies (n=82), 2020 to 2024 ........ 2

Supplementary Table S2. Overview of identified parameters related to COVID-19 in nursing homes with the corresponding references (n=82), 2020 to 2024 ........ 33

References of included studies (n=82), 2020 to 2024 ........ 37

**Supplementary Table S1** Characteristics and reported parameters of included studies (n=82), 2020 to 2024

| Author(s), publication year | Country | Study design | Nursing homes (n) | Participants (n) | Parameters | | | | |
|---|---|---|---|---|---|---|---|---|---|
| | | | | | Pandemic-related data | Facility level | Staff level | Resident level | Relative level |
| Aghili et al., 2022 (1) | Iran | Case-control study | 6 | 409 residents | • Preliminary signs and symptoms of SARS-CoV-2 infection (residents) | • Infection prevention and control principles<br>• Environmental and staff characteristics | | • Predisposing factors of SARS-CoV-2 infection | |
| Akdeniz et al., 2022 (2) | Turkey | Cross-sectional research design | 30 | 154 nursing home staff | | | • Workload<br>• Work stress | | |
| Akhtar-Danesh et al., 2022 (3) | Canada | Retrospective cohort study | - | 106,765 residents | | • Ownership | | • Index of neglect<br>• Mortality data<br>• Frailty index<br>• Changes in Health, Endstage Disease, Symptoms and Signs (CHESS) co-morbidity score | |
| Altintas et al., 2022 (4) | France | Survey | 4 | 129 nursing home staff | • Staff fears of COVID-19 | | • Emotional exhaustion<br>• Care quality experience | | |

| Author(s), publication year | Country | Study design | Nursing homes (n) | Participants (n) | Parameters | | | | |
|---|---|---|---|---|---|---|---|---|---|
| | | | | | Pandemic-related data | Facility level | Staff level | Resident level | Relative level |
| Arpacıoğlu et al., 2021 (5) | Turkey | Cross-sectional and descriptive design | 43 | 133 individuals (nursing home and community-dwelling) | | | | • Thinking and witnessing death, ambiguity of death, and pain<br>• Satisfaction with life<br>• Depression, anxiety, stress | |
| Backhaus et al., 2021 (6) | The Nether-lands | Mixed methods study (survey, documentation analyses) | 64 | - | • COVID-19 infections | • Dealing with infections | • Impact on well-being | • Visits, activities and daily life in care homes<br>• Impact on well-being | • Impact on well-being |
| Bielza et al., 2021 (7) | Spain | Retrospective cohort study | 55 | 630 residents with COVID-19 | • 30-day mortality, and associated factors<br>• Symptoms of a COVID-19 infection (dyspnea, consciousness)<br>• Risk of severe COVID-19 progression | • Hospitalisation rate of residents | | • Frailty | |

| Author(s), publication year | Country | Study design | Nursing homes (n) | Participants (n) | Parameters | | | | |
|---|---|---|---|---|---|---|---|---|---|
| | | | | | Pandemic-related data | Facility level | Staff level | Resident level | Relative level |
| Blain et al., 2020 (8) | France | Retrospective cohort study | 1 | 79 residents | • COVID-19 infections<br>• Symptoms of COVID-19 infections<br>• COVID-19 deaths | | | | |
| Blanco-Donoso et al., 2021 (9) | Spain | Cross-sectional study | - | 228 nursing home staff | • Staff fears of contagion | • Staff and personal protection equipment<br>• Co-worker and supervisor support | • Workload<br>• Social pressure from work<br>• Contact with death and suffering<br>• Secondary traumatic stress | | |
| Blanco-Donoso et al., 2022 (10) | Spain | Cross-sectional study | - | 335 nursing home staff | | | • Additionally:<br>• Emotional exhaustion<br>• Professional satisfaction<br>• Job demands and resources during the COVID-19 crisis | | |

| Author(s), publication year | Country | Study design | Nursing homes (n) | Participants (n) | Parameters | | | | |
|---|---|---|---|---|---|---|---|---|---|
| | | | | | Pandemic-related data | Facility level | Staff level | Resident level | Relative level |
| Brady et al., 2023 (11) | Ireland | Two cross-sectional surveys | 42 | Survey 1: 390 nursing home staff<br>Survey 2: 229 nursing home staff | • Staff perceptions: health fear, doubts about protective equipment, dissatisfaction with infection control-related systems and processes | | • Post-traumatic stress symptoms<br>• Wellbeing<br>• Suicidal ideation and planning<br>• Moral injury<br>• Coping styles<br>• Social isolation<br>• Job stress<br>• Altruistic acceptance of risk<br>• Work ability<br>• Needing support | | |
| Braun et al., 2020 (12) | USA | Cross-sectional study | 11,470 | - | • COVID-19 cases and deaths | • Ownership<br>• Staffing shortages<br>• Supplies of personal protective equipment | | • Deaths by any cause | |

| Author(s), publication year | Country | Study design | Nursing homes (n) | Participants (n) | Parameters | | | | |
|---|---|---|---|---|---|---|---|---|---|
| | | | | | Pandemic-related data | Facility level | Staff level | Resident level | Relative level |
| Bußmann & Pomorin, 2023 (13) | Germany | Longitudinal cohort study | 2 | Survey 1: 93 nursing home staff<br>Survey 2: 94 nursing home staff | | | • Psychosocial working conditions<br>• Psychosocial impact of dealing with residents receiving palliative care<br>• Psychosocial impact of contact with relatives<br>• COVID-19: organisation/ communication<br>• COVID-19: operational measures and overall assessment | | |
| Coffey et al., 2023 (14) | USA | One-time survey | 2 | 57 residents | • Feeling of safety from COVID-19 infection<br>• Risk reduction for severe course or death through vaccination | | | • Compulsory wearing of masks<br>• Desire for group activities<br>• Acceptance of COVID-19 tests and daily COVID-19 screening<br>• Quality of life | |

| Author(s), publication year | Country | Study design | Nursing homes (n) | Participants (n) | Parameters | | | | |
|---|---|---|---|---|---|---|---|---|---|
| | | | | | Pandemic-related data | Facility level | Staff level | Resident level | Relative level |
| Conejero et al., 2023 (15) | France | Cross-sectional study | 66 | 537 nursing home staff | | • Centre organisation<br>• Nursing home COVID-19 exposure | • Probable post-traumatic stress disorder<br>• Anxiety<br>• Depression<br>• Burnout of nursing home staff | | |
| De Souza Oliveira et al., 2023 (16) | Spain | Prospective study | 3 | 301 residents | | | | • Cognitive status<br>• Presence of chronic diseases and chronic treatments<br>• Frailty status<br>• Sarcopenia<br>• Functional status<br>• Nutritional status<br>• Anthropo-metrical and biochemical markers | |
| Dini et al., 2020 (17) | Italy | Cross-sectional study | 12 | 150 residents | • COVID-19 infections<br>• Symptoms of COVID-19 infections | | | | |

| Author(s), publication year | Country | Study design | Nursing homes (n) | Participants (n) | Parameters | | | | |
|---|---|---|---|---|---|---|---|---|---|
| | | | | | Pandemic-related data | Facility level | Staff level | Resident level | Relative level |
| Dora et al., 2020 (18) | USA | Prospective cohort study | 1 | 150 residents | • COVID-19 infections<br>• Symptoms of COVID-19 infections | | | | |
| Echeverria et al., 2020 (19) | Spain | Prospective cohort study | 169 | 10,347 residents | • COVID-19 infections<br>• COVID-19 mortality<br>• Symptoms of COVID-19 infections<br>• Number of staff isolated with suspected COVID-19 | | | • Mortality by any cause | |
| El Haj et al., 2020 (20) | France | Online survey | - | 58 residents | | | | • Anxiety<br>• Depression | |

| Author(s), publication year | Country | Study design | Nursing homes (n) | Participants (n) | Parameters | | | | |
|---|---|---|---|---|---|---|---|---|---|
| | | | | | Pandemic-related data | Facility level | Staff level | Resident level | Relative level |
| Estabrooks et al., 2023 (21) | Canada | Repeated cross-sectional survey | 35 | 181 managers | | | • Care Managers<br>• Mental health and well-being outcomes<br>• Job satisfaction<br>• Burnout<br>• Organizational citizenship behaviours<br>• Mental and physical health<br>• Burden of worry<br>• Intention to leave | | |
| Gao et al., 2022 (22) | China | Cross-sectional study | 4 | 381 residents | | | | • Status of depressive symptoms<br>• Physical function | |
| Gibson & Greene, 2020 (23) | USA | Cross-sectional study | 13,445 | - | | • Supply of personal protective equipment<br>• Staff shortage | | | |

| Author(s), publication year | Country | Study design | Nursing homes (n) | Participants (n) | Parameters | | | | |
|---|---|---|---|---|---|---|---|---|---|
| | | | | | **Pandemic-related data** | **Facility level** | **Staff level** | **Resident level** | **Relative level** |
| Graham et al., 2020 (24) | UK | Prevalence study | 4 | 394 residents<br>70 nursing home staff | • COVID-19 infections (residents and staff)<br>• Symptoms of COVID-19 infections | • Testing for COVID-19 infections among staff<br>• Testing for COVID-19 infection among residents | | • All-cause mortality | |
| Gustafsson et al., 2022 (25) | Sweden | Prospective national study | - | 11,782 residents | | | | • Perceived loneliness<br>• Self-rated health<br>• Worries and anxiety problems<br>• Mobility limitations | |
| Hering et al., 2022 (26) | Germany | Retrospective online survey | - | 811 nursing home staff | • COVID-19-related anxiety of staff<br>• COVID-19-related burden of staff | | • Working demands<br>• Satisfaction with COVID-19-related management<br>• Stress, anxiety, and depression<br>• Generalised anxiety disorder<br>• Patients health questionnaire<br>• Social relations at work | | |

| Author(s), publication year | Country | Study design | Nursing homes (n) | Participants (n) | Parameters | | | | |
|---|---|---|---|---|---|---|---|---|---|
| | | | | | Pandemic-related data | Facility level | Staff level | Resident level | Relative level |
| Hoben et al., 2023 (27) | Canada | Cross-sectional study | 9 | 689 residents | • COVID-19 outbreak | • Facility data | • Care unit-levels of care aide burnout<br>• Care aide data | • Quality of life<br>• Access to geriatric professionals | |
| Hoel et al., 2022 (28) | Germany | Cross-sectional study | - | 417 managers | • Cases of SARS-CoV-2 | • Structural characteristics | • Staff training to implement and use technology | • Social participation<br>• Behavioural and psychological symptoms of dementia<br>• Use of pharmaco-logical therapy | |
| Houben et al., 2023 (29) | The Nether-lands | Retrospective cohort study | 60 | - | • Community incidence<br>• Number of cases among staff | • Mechanical recirculation of air<br>• Ward size<br>• Providing psychogeriatric care<br>• Restrictions on staff movement between wards and facilities | | | |

| Author(s), publication year | Country | Study design | Nursing homes (n) | Participants (n) | Parameters | | | | |
|---|---|---|---|---|---|---|---|---|---|
| | | | | | Pandemic-related data | Facility level | Staff level | Resident level | Relative level |
| Husky et al., 2022 (30) | France | Cross-sectional online survey | 6 | 127 nursing home staff | • COVID-19 infection status of staff<br>• Staff experiences during lockdown; COVID-19-related fears | | • Probable generalised anxiety<br>• Panic attacks<br>• Depression<br>• Posttraumatic stress<br>• Substance use disorders<br>• Prior mental health problems | | |
| Khairat et al., 2021 (31) | USA | Cross-sectional study | 14,690 | - | • Spread of COVID-19 within the nursing home's neighbourhood | • Star rating<br>• Case mix<br>• For-profit status<br>• Bed size | | | |
| Kittang et al., 2020 (32) | Norway | Retrospective observational study | 3 | - | • COVID-19 infections (residents and staff)<br>• Symptoms of COVID-19 infections<br>• Mortality associated with COVID-19<br>• Staff quarantine | • Hospitalisation rate of residents | | • Recovery | |

| Author(s), publication year | Country | Study design | Nursing homes (n) | Participants (n) | Parameters | | | | |
|---|---|---|---|---|---|---|---|---|---|
| | | | | | Pandemic-related data | Facility level | Staff level | Resident level | Relative level |
| Kühl et al., 2022 (33) | Germany | Retrospective online survey | 1,010 | 1,010 managers | • COVID-19 cases<br>• COVID-19 incidence on the state level | • General characteristics of nursing homes<br>• COVID-19 impact on general practitioners' care<br>• Visiting restrictions for general practitioners<br>• Deficits in general practitioners' care | • COVID-19-related burden by nursing home managers | | |
| Ladhani et al., 2020 (34) | UK | Cohort study | 6 | 264 residents 254 nursing home staff | • COVID-19 infections (residents and staff)<br>• Symptoms of COVID-19 infections<br>• Mortality associated with COVID-19 | • Hospitalisation rate of residents | | • Recovery | |

| Author(s), publication year | Country | Study design | Nursing homes (n) | Participants (n) | Parameters | | | | |
|---|---|---|---|---|---|---|---|---|---|
| | | | | | Pandemic-related data | Facility level | Staff level | Resident level | Relative level |
| Levere et al., 2021 (35) | USA | Quantitative analysis of resident-level assessment data | 224 | 29,097 residents | | | | • Any depressive symptoms<br>• Weight loss<br>• Pressure ulcer<br>• Incontinence<br>• Cognitive functioning<br>• Activities of daily living | |
| Li et al., 2021 (36) | USA | Cross-sectional study | 14,062 | - | • Resident and staff COVID-19 cases | • Ownership<br>• Supply of personal protective equipment | | | |
| Lipsitz et al., 2020 (37) | USA | Longitudinal cohort study | 360 | - | • COVID-19 infections (residents and staff)<br>• Mortality associated with COVID-19<br>• County COVID-19 prevalence | • Hospitalisation rate of residents | | | |
| Louie et al., 2021 (38) | USA | Cross-sectional study | 4 | - | • COVID-19 infections (residents and staff)<br>• Mortality associated with COVID-19 | • Testing for COVID-19 infections among staff and residents<br>• Hospitalisation rate of residents | | | |

| Author(s), publication year | Country | Study design | Nursing homes (n) | Participants (n) | Parameters | | | | |
|---|---|---|---|---|---|---|---|---|---|
| | | | | | Pandemic-related data | Facility level | Staff level | Resident level | Relative level |
| Marossy et al., 2021 (39) | UK | Cross-sectional study | 37 | 2,455 residents and nursing home staff | • COVID-19 infections (residents and staff)<br>• Symptoms of COVID-19 infections | • Testing for COVID-19 infections among staff and residents | | | |
| Martinchek et al., 2021 (40) | USA | Retrospective chart review | 1 | 209 residents | | | | • Weight loss | |
| McArthur et al., 2021 (41) | Canada | Retrospective chart review | 7 | 765 residents | | | | • Depression<br>• Delirium<br>• Behavioural problems | |
| McConeghy et al., 2020 (42) | USA | Retrospective cohort study | 416 | 4,669 residents | • COVID-19 infections (residents) | • Testing for COVID-19 infections among residents | | | |
| McDermid et al., 2023 (43) | UK | Comparative analysis of baseline data from two studies | S1: 69<br>S2: 149 | S1: 1006 residents<br>S2: 666 residents | | • Size (total number of residents; number of residents with dementia)<br>• Staffing information | | • Antipsychotic prescriptions<br>• Neuro-psychiatric symptoms | |

| Author(s), publication year | Country | Study design | Nursing homes (n) | Participants (n) | Parameters | | | | |
|---|---|---|---|---|---|---|---|---|---|
| | | | | | Pandemic-related data | Facility level | Staff level | Resident level | Relative level |
| McGarry et al., 2020 (44) | USA | Cohort study | 15,035 | - | • Resident and staff COVID-19 cases | • Shortage of personal protective equipment<br>• Shortage of staff<br>• Case mix<br>• Quality score | | | |
| Monin et al., 2020 (45) | USA | Cross-sectional survey | - | 161 relatives | | | | • Positive emotions<br>• Negative emotions | • Communication methods other than physical visits (e.g., phone, video-conference, e-mail, and letters)<br>• Positive emotions<br>• Negative emotions |

| Author(s), publication year | Country | Study design | Nursing homes (n) | Participants (n) | Parameters | | | | |
|---|---|---|---|---|---|---|---|---|---|
| | | | | | Pandemic-related data | Facility level | Staff level | Resident level | Relative level |
| Montoya et al., 2021 (46) | USA | Retrospective cohort study | 3 | 215 residents | • COVID-19 infections<br>• Mortality associated with COVID-19<br>• Symptoms of COVID-19 infections<br>• Risk of severe COVID-19 progression | • Testing for COVID-19 infections among residents<br>• Hospitalisation rate of residents<br>• Pandemic-related control measures: cohorting of COVID-19 positive residents; personal protective equipment reeducation, and use | | | |
| Nash et al., 2021 (47) | USA | Cross-sectional survey | - | 512 relatives | | | | | • Sadness<br>• Trauma<br>• Anger<br>• Frustration<br>• Helplessness<br>• Anxiety<br>• Four overarching themes: 1) isolation 2) rapid decline 3) inhumane care and 4) lack of oversight |

| Author(s), publication year | Country | Study design | Nursing homes (n) | Participants (n) | Parameters | | | | |
|---|---|---|---|---|---|---|---|---|---|
| | | | | | Pandemic-related data | Facility level | Staff level | Resident level | Relative level |
| Navarro Prados et al., 2022 (48) | Spain | Cross-sectional survey | - | 340 nursing home staff | • Organisational characteristics of the job related to COVID-19 | | • Sense of Coherence<br>• Burnout syndrome | | |
| Navarro Prados et al., 2024 (49) | Spain | Cross-sectional survey | - | 165 nursing home staff | | | • Professional characteristics<br>• Burnout<br>• Resilience<br>• Experiential avoidance and psychological inflexibility<br>• Satisfaction with life<br>• Depression | | |
| O'Caoimh et al., 2020 (50) | Ireland | Cross-sectional survey | - | 202 relatives | | | | | • Loneliness<br>• Psychological well-being<br>• Quality of life<br>• Satisfaction with care<br>• Perception of staff support |
| Oliveira et al., 2023 (51) | Spain | Prospective study | 3 | 301 residents<br>119 nursing home staff<br>51 relatives | | | • Anxiety<br>• Depression<br>• Perception about the meaning of suffering<br>• Burnout status | • Anxiety<br>• Depression<br>• Perception about the meaning of suffering<br>• Social support | |

| Author(s), publication year | Country | Study design | Nursing homes (n) | Participants (n) | Parameters | | | | |
|---|---|---|---|---|---|---|---|---|---|
| | | | | | Pandemic-related data | Facility level | Staff level | Resident level | Relative level |
| Patel et al., 2020 (52) | USA | Prospective cohort study | 1 | 126 residents | • COVID-19 infections<br>• Mortality associated with COVID-19<br>• Symptoms of COVID-19 infections | • Testing for COVID-19 infections among residents<br>• Hospitalisation | | | |
| Pérez-Rodríguez et al., 2021 (53) | Spain | Observational study | 4 | 435 residents | | | | • Clinical data<br>• Functional, cognitive, and nutritional data<br>• Mental status<br>• Depression<br>• Body mass index | |

| | | | | | | | | | |
|---|---|---|---|---|---|---|---|---|---|
| Pförtner et al., 2021 (54) | Germany | Cross-sectional study | - | 299 managers (outpatient and inpatient long-term care facilities) | • Concerns about SARS-CoV-2 infections among residents and employees | • Infection control equipment<br>• Compliance with hygiene guidelines<br>• Testing for SARS-CoV-2 infection (patients, employees)<br>• Isolation of SARS-CoV-2 infected patients<br>• Compliance with contact restrictions for relatives<br>• Work-related information for care facilities and employees<br>• Provision of funding to address the pandemic<br>• General demands: staff shortage, staff overload, compliance with regulations on working hours, compliance with staffing ratio, loss of income, high intensity and | | | |

| Author(s), publication year | Country | Study design | Nursing homes (n) | Participants (n) | Parameters | | | | |
|---|---|---|---|---|---|---|---|---|---|
| | | | | | Pandemic-related data | Facility level | Staff level | Resident level | Relative level |
| | | | | | | density of work, implementation of service meetings, fear for the well-being of patients, image, family doctor care, availability of external staff, expectations of relatives | | | |
| Preuß et al., 2022 (55) | Germany | Two cross-sectional surveys | 1,067 | - | • General spread<br>• Residents deaths | • Number of residents<br>• Number of staff<br>• Nursing home characteristics | | | |
| Prins et al., 2021 (56) | The Nether-lands | Cross-sectional study | - | 958 relatives | | | | | • Contact frequency<br>• Resilience<br>• Worries about the person with dementia<br>• Loneliness |

| Author(s), publication year | Country | Study design | Nursing homes (n) | Participants (n) | Parameters | | | | |
|---|---|---|---|---|---|---|---|---|---|
| | | | | | Pandemic-related data | Facility level | Staff level | Resident level | Relative level |
| Queiroz et al., 2023 (57) | Brazil | Prospective cohort study | 13 | 289 residents | • Covid-19 diagnosis | | | • Lifestyle assessment<br>• Clinical Assessment<br>• Anthropometric Assessment<br>• Functional Assessment | |
| Rawle et al., 2020 (58) | UK | Retrospective cohort study | - | 64 residents | • Symptoms of COVID-19 infections<br>• Mortality associated with COVID-19<br>• Risk of severe COVID-19 progression | • Lengths of hospital stay | | • Frailty<br>• Anorexia | |
| Riello et al., 2020 (59) | Italy | Online survey | 33 | 1,071 nursing home staff | | | • Anxiety<br>• Post-traumatic symptoms | | |

| Author(s), publication year | Country | Study design | Nursing homes (n) | Participants (n) | Parameters | | | | |
|---|---|---|---|---|---|---|---|---|---|
| | | | | | Pandemic-related data | Facility level | Staff level | Resident level | Relative level |
| Rojo-Perez et al., 2022 (60) | Spain | Secondary data analysis (survey) | - | 447 residents | | | | • Perception of the residential environment<br>• Perception of safety and management<br>• Mobility<br>• Personal situation with regard to the pandemic<br>• Objective physical health<br>• Self-perception of health<br>• Mental health<br>• Feelings and coping<br>• Performance of leisure and participation activities<br>• Quality of life<br>• Self-perception of ageing | |

| Author(s), publication year | Country | Study design | Nursing homes (n) | Participants (n) | Parameters | | | | |
|---|---|---|---|---|---|---|---|---|---|
| | | | | | Pandemic-related data | Facility level | Staff level | Resident level | Relative level |
| Rutten et al., 2020 (61) | The Nether-lands | Prospective cohort study | - | 4,007 residents with clinically suspected COVID-19 | • COVID-19 infections<br>• Symptoms of COVID-19 infections<br>• Mortality associated with COVID-19<br>• Risk of severe COVID-19 progression | | | | |
| Ryskina et al., 2021 (62) | USA | Cross-sectional study | 11,585 | - | • COVID-19 cases among staff<br>• Local COVID-19 prevalence<br>• Confirmed resident cases | • Number of beds<br>• Ownership<br>• Shortage of personal protective equipment | | | |

| Author(s), publication year | Country | Study design | Nursing homes (n) | Participants (n) | Parameters | | | | |
|---|---|---|---|---|---|---|---|---|---|
| | | | | | Pandemic-related data | Facility level | Staff level | Resident level | Relative level |
| Sacco et al., 2020 (63) | France | Retrospective cohort study | 1 | 87 residents<br>92 nursing home staff | • COVID-19 infections (residents and staff)<br>• Symptoms of COVID-19 infections (residents)<br>• Mortality associated with COVID-19 (residents)<br>• Risk of severe COVID-19 progression (residents) | • Testing for COVID-19 infections among residents and staff | | • Falls<br>• Altered consciousness<br>• Anorexia | |
| Savci et al., 2021 (64) | Turkey | Descriptive and correlational study | 1 | 103 residents | | | | • Global cognition Mini Mental State Exam<br>• Resilience<br>• Loneliness<br>• Quality of Life<br>• Fear of COVID-19 | |
| Schulze et al., 2022 (65) | Germany | Cross-sectional study with quantitative and qualitative data | 37 | 177 nursing home staff | | | • Psychosocial burden at work | | |

| Author(s), publication year | Country | Study design | Nursing homes (n) | Participants (n) | Parameters | | | | |
|---|---|---|---|---|---|---|---|---|---|
| | | | | | Pandemic-related data | Facility level | Staff level | Resident level | Relative level |
| Senczyszyn et al., 2020 (66) | Poland | Online survey | - | 178 nursing home staff | | • Availability of personal protective equipment<br>• Safety guidelines | • General health (somatic symptoms, anxiety, insomnia, social dysfunction, depression)<br>• Access to psychiatric and psychological support at the workplace | • Mood<br>• Cognition,<br>• Dependency<br>• Dealing with COVID-19 restrictions | |
| Shi et al., 2020 (67) | USA | Retrospective cohort study | 1 | 389 residents | • COVID-19 infections<br>• Symptoms of COVID-19 infections<br>• Mortality associated with COVID-19<br>• Risk of severe COVID-19 progression<br>• Staff residence | | | • Frailty<br>• Delirium<br>• Anorexia | |

| Author(s), publication year | Country | Study design | Nursing homes (n) | Participants (n) | Parameters | | | | |
|---|---|---|---|---|---|---|---|---|---|
| | | | | | Pandemic-related data | Facility level | Staff level | Resident level | Relative level |
| Takahashi et al., 2022 (68) | Japan | Large-scale web-based survey | 284 | 676 nursing home staff | • Infections at facilities | • Infection protection<br>• Changes in nursing home users<br>• Cooperation with other medical institutions<br>• Prejudice/ discrimination | • Depression and anxiety | | |
| Tang et al., 2020 (69) | USA | Retrospective cohort study | 15 | 1,970 residents | • COVID-19 infections<br>• Symptoms of COVID-19 infections<br>• Mortality associated with COVID-19<br>• Risk of severe COVID-19 progression | • Hospitalisation | | | |
| Tebbeb et al., 2022 (70) | France | Qualitative and quantitative survey | 12 | 373 nursing home staff | | | • Experience of the COVID-19 crisis<br>• Perceived stress<br>• Anxiety/depres sive symptoms<br>• Post-traumatic stress disorder | | |

| Author(s), publication year | Country | Study design | Nursing homes (n) | Participants (n) | Parameters | | | | |
|---|---|---|---|---|---|---|---|---|---|
| | | | | | Pandemic-related data | Facility level | Staff level | Resident level | Relative level |
| Telford et al., 2021 (71) | USA | Evaluation study | 24 | 2,580 residents | • Infection prevalence | • Infection prevention and control adherence to current guidelines for key indicators across five categories: hand hygiene, disinfection, social distancing, personal protective equipment and symptom screening | | | |

| Author(s), publication year | Country | Study design | Nursing homes (n) | Participants (n) | Parameters | | | | |
|---|---|---|---|---|---|---|---|---|---|
| | | | | | **Pandemic-related data** | **Facility level** | **Staff level** | **Resident level** | **Relative level** |
| Urlings et al., 2023 (72) | The Nether-lands | Cross-sectional survey | 59 | 59 managers or quality officers | • Infections in the nursing home<br>• Progress of the vaccination campaign of residents and staff<br>• Effects of vaccination on the daily lives of staff<br>• Effect of the prolonged COVID-19 pandemic and protective restrictions on staff<br>• Effects of vaccination on the daily lives of residents | • Compliance with protective measures<br>• Plans to further relax protective measures<br>• Pressure and support experienced by nursing homes | • Sick leave and experienced work pressure by staff | • Effect of the prolonged COVID-19 pandemic and protective restrictions on residents | • Effect of the prolonged COVID-19 pandemic and protective restrictions on family members |
| van der Roest et al., 2020 (73) | The Nether-lands | Online survey | - | 193 residents<br>623 nursing home staff<br>1,387 relatives | | | | • Loneliness<br>• Mood<br>• Happiness<br>• Sadness<br>• Agitation<br>• Depression<br>• Anxiety<br>• Irritability<br>• Appetite disorders | |

| Author(s), publication year | Country | Study design | Nursing homes (n) | Participants (n) | Parameters | | | | |
|---|---|---|---|---|---|---|---|---|---|
| | | | | | Pandemic-related data | Facility level | Staff level | Resident level | Relative level |
| van Dijk et al., 2022 (74) | The Nether-lands | Cross-sectional multilevel study | 10 | 1,669 nursing home staff | • COVID-19 contact, infections and worries (staff) | | • Job demands<br>• Work functioning<br>• Depressive symptoms<br>• Burnout | | |
| Vandael et al., 2022 (75) | Belgium | COVID-19 surveillance | 1,542 | - | • Residents with COVID-19 infection<br>• COVID-19 deaths<br>• Staff with COVID-19 infections | • Nursing home characteristics<br>• Hospitalizations due to COVID-19 | | | |
| Verbeek et al., 2020 (76) | The Nether-lands | Mixed methods study | 26 | 26 managers, quality or policy officers, nursing home staff | | • Context of the nursing home<br>• Application of national guidelines | • Well-being | • Visits<br>• Well-being | • Compliance with local protocol<br>• Well-being |
| von der Warth et al., 2022 (77) | Germany | Cross-sectional study | 40 | 419 residents | | | | • Quality of life<br>• Self-rated nursing care satisfaction<br>• Medical care satisfaction<br>• Visits of relatives | |

| Author(s), publication year | Country | Study design | Nursing homes (n) | Participants (n) | Parameters | | | | |
|---|---|---|---|---|---|---|---|---|---|
| | | | | | Pandemic-related data | Facility level | Staff level | Resident level | Relative level |
| Wammes et al., 2020 (78) | The Nether-lands | Cross-sectional survey | - | 1,997 relatives | | | | | • Perspectives regarding nursing home visiting restrictions<br>• Communication access to residents<br>• Adverse effects of visiting restrictions<br>• Potential protective effect of visiting restrictions<br>• Important aspects for relatives during and after visiting restrictions |
| Watts et al., 2023 (79) | USA | Quarterly cross-sectional data from Minimum Data Sets | - | 96 residents | | | | • Cognitive function<br>• Transfer ability<br>• Depression<br>• Rejecting care<br>• Falls<br>• Urinary tract infections<br>• Physical conflict<br>• Verbal conflict | |

| Author(s), publication year | Country | Study design | Nursing homes (n) | Participants (n) | Parameters | | | | |
|---|---|---|---|---|---|---|---|---|---|
| | | | | | Pandemic-related data | Facility level | Staff level | Resident level | Relative level |
| Xu et al., 2020 (80) | USA | Cross-sectional study | 11,920 | - | • Resident and staff COVID-19 cases | • Availability of personal protective equipment<br>• Shortage of staff<br>• Case mix | | | |
| Yan et al., 2023 (81) | USA | Observational study based on the Minimum Data Set and Medicare claims | 15,751 | 2,787,961 residents | | • Certified nursing assistants' staffing hours per bed per day in a quarter<br>• Facility-level characteristics | | • Antipsychotic use during a quarter<br>• Aggressive behaviour<br>• Cognitive impairment | |
| Ye et al., 2021 (82) | USA | Retrospective chart review | 15 | 963 residents | | • Dealing with residents within Advanced Care Planning | | • Care preference changes/ changes of personal goals of care within Advanced Care Planning | |

**Supplementary Table S2** Overview of identified parameters related to COVID-19 in nursing homes with the corresponding references (n=82), 2020 to 2024

| Identified parameters | References |
|---|---|
| **Pandemic-related data** | |
| Pandemic progression: | |
| Local COVID-19 incidence/prevalence | (29, 31, 33, 37, 55, 62, 67) |
| COVID-19 outbreak/infection rate in nursing home (number of residents) | (6, 8, 12, 17-19, 24, 27, 28, 32-34, 36-39, 42, 44, 46, 52, 57, 61-63, 67-69, 71, 72, 75, 80) |
| COVID-19 outbreak/infections among staff/staff absences | (19, 24, 29, 30, 32, 34, 36-39, 44, 62, 63, 74, 75, 80) |
| Progress in the vaccination campaign for residents and staff | (72) |
| Staff level: | |
| Symptoms of a COVID-19 infection | (24, 34, 38, 39) |
| Staff fears and concerns (... of infecting themselves, residents, or their relatives) | (4, 9, 11, 26, 54, 74) |
| Impact of vaccination on the daily lives of staff | (72) |
| Impact of the prolonged COVID-19 pandemic and protective restrictions on staff | (26, 30, 48, 72) |
| Resident level: | |
| Risk of severe COVID-19 progression (especially male gender, age, chronic renal insufficiency, dementia, haematological/oncological diseases) | (7, 46, 58, 61, 63, 67, 69) |
| Symptoms of a COVID-19 infection | (1, 7, 8, 14, 17-19, 24, 32, 34, 39, 46, 52, 58, 61, 63, 67, 69) |
| Mortality related to COVID-19 | (7, 8, 12, 32, 34, 37, 38, 46, 52, 55, 58, 61, 63, 67, 69, 75) |
| Impact of vaccination on the daily lives of residents | (14, 72) |
| **Facility level: epidemiological and general data** | |
| Nursing home characteristics: | |
| Nursing home size/number of beds/number of residents | (28, 31, 33, 43, 55, 62, 75, 76, 81) |
| Size of the care units | (29) |
| Ownership of the nursing home | (3, 12, 28, 31, 36, 55, 62, 76, 81) |
| Specialised units | (28, 29, 55) |
| Case mix | (31, 44, 80) |
| Separate sleeping areas/single-occupancy or shared rooms | (64, 76) |
| Nursing home quality of care | (31, 44) |
| Staff deployment: | |
| Number of staff/staffing ratio/staff shortages | (1, 9, 12, 23, 28, 43, 44, 54, 55, 75, 80, 81) |
| Staffing plan or adjustment of staff assignment (assignment of tasks/changes to the scope of work/changes to processes and procedures/formation of stable teams/availability of external staff/absence rules for staff with COVID-19 infection) | (1, 54, 76) |
| Feeling of being well informed and advised | (54) |
| Burdens on the management level | (21, 33) |

| | |
|---|---|
| **Facility level: pandemic-related protective and control measures** | |
| Use of air filters | (29) |
| Contact reduction (e.g. visiting bans/access restrictions for relatives/volunteers/external service providers) | (71) |
| Change in contact options (tablet, telephone, digital communication)/offer of alternatives: communication through window panes, outdoor events, hygiene protection wall/separators | (28, 78) |
| Cancellation of social activities | (28) |
| Cohorting/relocation/transfer/isolation of COVID-19 infected residents within the facility | (46, 54) |
| Testing for COVID-19 infections among staff | (24, 38, 39, 54, 63) |
| Testing for COVID-19 infections among residents | (24, 38, 39, 42, 46, 52, 54, 63) |
| Provision of hygiene material and personal protective equipment | (9, 12, 23, 36, 44, 46, 54, 62, 66, 76, 80) |
| Implementation of hygiene rules or information from public authorities/pandemic plan | (54, 56, 66, 71, 72, 76) |
| Training of nursing staff (protective clothing, special hygiene instructions, dealing with highly infectious residents) | (46, 71) |
| **Facility level: nursing and health care** | |
| Access restrictions for general practitioners and medical specialists | (33) |
| General practitioners' or medical specialists' care deficit (in routine or acute cases) | (33, 54) |
| Acute inpatient care (hospitalisation rate of residents) | (7, 32, 34, 37, 38, 46, 52, 69, 75) |
| End-of-life care/dealing with residents in palliative situations | (82) |
| Cooperation with external institutions or persons | (27, 54, 68) |
| **Staff level** | |
| Psychosocial factors at the workplace: | |
| Impact of changing work requirements and flexibility requirements | (10, 26, 65, 74) |
| Work intensity and work intensification | (2, 9, 54, 65, 72) |
| Workload | (2, 26) |
| Psychosocial effects of dealing with palliative care residents | (9, 13, 51) |
| Uncertainties regarding the implementation of frequently changing regulations and guidelines (sometimes daily-changing rules), role ambiguities | (48, 54, 65, 74) |
| Needs of nursing staff (e.g. recognition and appreciation of care, coronavirus bonus, supervision for nursing staff during the pandemic) | (9, 11, 26, 48, 66) |
| Psychosocial effects of interacting with relatives | (13) |
| Considerations of changing or leaving the profession | (21, 65) |
| Job satisfaction | (2, 10, 21, 65) |
| Satisfaction with the quality of nursing care | (4) |
| Effects on the general health status | (11, 65, 66) |
| Burnout | (9, 10, 15, 21, 27, 48, 49, 51, 65, 74) |
| Effects on presenteeism | (65) |
| Personal burden, stress, anxiety, depression, fatigue, panic attacks, helplessness, loss of control | (2, 9, 11, 15, 26, 30, 49, 51, 59, 65, 66, 68, 70, 74) |
| Physical and mental overload of staff | (4, 11, 48) |
| Substance abuse | (30) |

| | |
|---|---|
| Suicidal thoughts and plans | (11) |
| Experiences of prejudice and discrimination | (54, 68) |
| Staff well-being | (6, 11, 21, 49) |
| Fear for the well-being of the residents | (54) |
| Coping strategies, resilience | (11, 48, 49) |
| **Resident level** | |
| General health status: | |
| Functional status | (16, 22, 53, 57, 60) |
| Nutritional status (body mass index) | (16, 35, 40, 53, 57, 58, 63, 67) |
| Frailty | (3, 7, 16, 58, 67) |
| Cognition and orientation/delirium | (16, 35, 41, 53, 63, 64, 66, 67, 79, 81) |
| Participation: | |
| Visits (number, location and visitors) | (6, 56, 76, 77) |
| Relationships with significant others and loss of significant others (staff, residents, family, friends) | (56, 60) |
| Possibility of social participation/external participation | (28, 56, 60) |
| Possibility of internal participation | (14) |
| Social isolation, social distancing and loneliness | (60, 64, 73) |
| Physical and psychological effects: | |
| Mood and enjoyment of life | (45, 66, 73) |
| Sadness, listlessness | (45, 73) |
| Depression/depressive symptoms | (5, 20, 22, 25, 35, 41, 51, 53, 60, 73, 79) |
| Anxiety and stress | (5, 20, 25, 51, 64, 73) |
| COVID-19-related anxiety and coping strategies/resilience | (60, 64) |
| Behaviour and psychiatric symptoms of dementia (BPSD)/psychotropic drugs | (28, 41, 43, 73, 81) |
| Sleep problems | (64) |
| Quality of life and well-being | (5, 6, 14, 25, 27, 60, 64, 77) |
| Need for support | (51) |
| Dealing with protective measures/sense of security/requests | (14, 60, 66) |
| Satisfaction with nursing and health care | (77) |
| Recovery | (32, 34) |
| All-cause mortality | (3, 12, 19, 24) |
| Evaluation of nursing care: | |
| Mobility/falls | (25, 63, 79) |
| Change in the general need for care | (35, 66) |
| Neglect (decubitus, dehydration, urinary tract infection) | (3, 35, 79) |
| Pain | (5) |
| Effects on health care provision | (82) |
| Dealing with dying and death | (5, 51, 82) |

| **Relative level** | |
|---|---|
| Relatives' expectations | (54) |
| Ways of communication with the facility and residents | (45, 78) |
| Perception of the health care situation | (47, 50, 56) |
| Support needs of relatives | (50) |
| Experience and acceptance of protective measures/sense of security/requests | (47, 56, 72, 76, 78) |
| Compliance with local visitation and hygiene protocol and its implications | (54, 72) |
| Perceived stressful situation of the relatives (feelings of anger and annoyance/feelings of despair/emotionally stressful conflicts/feelings of helplessness/sadness/frustration/fear/trauma/resilience) | (45, 47, 56, 78) |
| Loneliness/social isolation/social distancing | (50, 56, 78) |
| Quality of life and well-being of relatives | (6, 45, 50) |

## References of included studies (n=82), 2020 to 2024


1. Aghili MS, Darvishpoor Kakhki A, Gachkar L, Davidson PM. Predictors of contracting COVID-19 in nursing homes: implications for clinical practice. J Adv Nurs. 2022;78(9):2799-806.
2. Akdeniz Ş, Çoban M, Koç O, Pekesen M, Korkmaz Yaylagul N, Sönmez S, et al. Determination of workload, work stress and related factors in nursing home workers during the COVID-19 pandemic in Turkey. Int J Environ Res Public Health. 2022;20(1).
3. Akhtar-Danesh N, Baumann A, Crea-Arsenio M, Antonipillai V. Frequency of neglect and its effect on mortality in long-term care before and during the COVID-19 pandemic. Healthc Policy. 2022;17(Sp):107-21.
4. Altintas E, Boudoukha AH, Karaca Y, Lizio A, Luyat M, Gallouj K, et al. Fear of COVID-19, emotional exhaustion, and care quality experience in nursing home staff during the COVID-19 pandemic. Arch Gerontol Geriatr. 2022;102:104745.
5. Arpacıoğlu S, Yalçın M, Türkmenoğlu F, Ünübol B, Çelebi Çakıroğlu O. Mental health and factors related to life satisfaction in nursing home and community-dwelling older adults during COVID-19 pandemic in Turkey. Psychogeriatrics. 2021;21(6):881-91.
6. Backhaus R, Verbeek H, de Boer B, Urlings JHJ, Gerritsen DL, Koopmans R, et al. From wave to wave: a Dutch national study on the long-term impact of COVID-19 on well-being and family visitation in nursing homes. BMC Geriatr. 2021;21(1):588.
7. Bielza R, Sanz J, Zambrana F, Arias E, Malmierca E, Portillo L, et al. Clinical characteristics, frailty, and mortality of residents with COVID-19 in nursing homes of a region of Madrid. J Am Med Dir Assoc. 2021;22(2):245-52 e2.
8. Blain H, Rolland Y, Benetos A, Giacosa N, Albrand M, Miot S, et al. Atypical clinical presentation of COVID-19 infection in residents of a long-term care facility. Eur Geriatr Med. 2020;11(6):1085-8.
9. Blanco-Donoso LM, Moreno-Jiménez J, Amutio A, Gallego-Alberto L, Moreno-Jiménez B, Garrosa E. Stressors, job resources, fear of contagion, and secondary traumatic stress among nursing home workers in face of the COVID-19: the case of Spain. J Appl Gerontol. 2021;40(3):244-56.
10. Blanco-Donoso LM, Moreno-Jiménez J, Gallego-Alberto L, Amutio A, Moreno-Jiménez B, Garrosa E. Satisfied as professionals, but also exhausted and worried: the role of job demands, resources and emotional experiences of Spanish nursing home workers during the COVID-19 pandemic. Health Soc Care Community. 2022;30(1):e148-e60.
11. Brady C, Shackleton E, Fenton C, Loughran O, Hayes B, Hennessy M, et al. Worsening of mental health outcomes in nursing home staff during the COVID-19 pandemic in Ireland. PLoS One. 2023;18(9):e0291988.
12. Braun RT, Yun H, Casalino LP, Myslinski Z, Kuwonza FM, Jung HY, et al. Comparative performance of private equity-owned US nursing homes during the COVID-19 pandemic. JAMA Netw Open. 2020;3(10):e2026702.
13. Bußmann A, Pomorin N. Psychosocial burdens in palliative care: a longitudinal cohort study in nursing homes and impacts of the COVID-19 pandemic. BMC Palliat Care. 2023;22(1):163.
14. Coffey KC, Lydecker AD, Roghmann MC. What did nursing home residents think about COVID-19 prevention practices in the era of vaccination and treatment? Am J Infect Control. 2023;51(12):1452-4.
15. Conejero I, Petrier M, Fabbro Peray P, Voisin C, Courtet P, Potier H, et al. Post-traumatic stress disorder, anxiety, depression and burnout in nursing home staff in South France during the COVID-19 pandemic. Transl Psychiatry. 2023;13(1):205.
16. De Souza Oliveira AC, Gómez Gallego M, Martínez CG, López Mongil R, Moreno Molina J, Hernández Morante JJ, et al. Effects of COVID-19 lockdown on nutritional, functional and frailty biomarkers of people living in nursing homes. A prospective study. Biol Res Nurs. 2023;25(4):615-26.
17. Dini FL, Bergamini C, Allegrini A, Scopelliti M, Secco G, Miccoli M, et al. Bedside wireless lung ultrasound for the evaluation of COVID-19 lung injury in senior nursing home residents. Monaldi Arch Chest Dis. 2020;90(3).
18. Dora AV, Winnett A, Jatt LP, Davar K, Watanabe M, Sohn L, et al. Universal and serial laboratory testing for SARS-CoV-2 at a long-term care skilled nursing facility for veterans - Los Angeles, California, 2020. MMWR Morb Mortal Wkly Rep. 2020;69(21):651-5.

19. Echeverria P, Mas Bergas MA, Puig J, Isnard M, Massot M, Vedia C, et al. COVIDApp as an innovative strategy for the management and follow-up of COVID-19 cases in long-term care facilities in Catalonia: implementation study. JMIR Public Health Surveill. 2020;6(3):e21163.
20. El Haj M, Altintas E, Chapelet G, Kapogiannis D, Gallouj K. High depression and anxiety in people with Alzheimer's disease living in retirement homes during the Covid-19 crisis. Psychiatry Res. 2020;291:113294.
21. Estabrooks CA, Duan Y, Cummings GG, Doupe M, Hoben M, Keefe J, et al. Changes in health and well-being of nursing home managers from a prepandemic baseline in February 2020 to December 2021. J Am Med Dir Assoc. 2023;24(2):148-55.
22. Gao L, Yang J, Liu J, Xin T, Liu Y. Depressive symptoms and physical function among the elderly in nursing homes during the COVID-19 pandemic in China: a cross-sectional study. Medicine (Baltimore). 2022;101(47):e31929.
23. Gibson DM, Greene J. State actions and shortages of personal protective equipment and staff in U.S. nursing homes. J Am Geriatr Soc. 2020;68(12):2721-6.
24. Graham NSN, Junghans C, Downes R, Sendall C, Lai H, McKirdy A, et al. SARS-CoV-2 infection, clinical features and outcome of COVID-19 in United Kingdom nursing homes. J Infect. 2020;81(3):411-9.
25. Gustafsson PE, Schröders J, Nilsson I, San Sebastián M. Surviving through solitude: a prospective national study of the impact of the early COVID-19 pandemic and a visiting ban on loneliness among nursing home residents in Sweden. J Gerontol B Psychol Sci Soc Sci. 2022;77(12):2286-95.
26. Hering C, Gangnus A, Budnick A, Kohl R, Steinhagen-Thiessen E, Kuhlmey A, et al. Psychosocial burden and associated factors among nurses in care homes during the COVID-19 pandemic: findings from a retrospective survey in Germany. BMC Nurs. 2022;21(1):41.
27. Hoben M, Dymchuk E, Corbett K, Devkota R, Shrestha S, Lam J, et al. Factors associated with the quality of life of nursing home residents during the COVID-19 pandemic: a cross-sectional study. J Am Med Dir Assoc. 2023;24(6):876-84.e5.
28. Hoel V, Seibert K, Domhoff D, Preuß B, Heinze F, Rothgang H, et al. Social health among German nursing home residents with dementia during the COVID-19 pandemic, and the role of technology to promote social participation. Int J Environ Res Public Health. 2022;19(4).
29. Houben F, den Heijer CDJ, Dukers-Muijrers NHTM, Daamen AMJ, Groeneveld NS, Vijgen GCM, et al. Facility- and ward-level factors associated with SARS-CoV-2 outbreaks among residents in long-term care facilities: a retrospective cohort study. Int J Infect Dis. 2023;130:166-75.
30. Husky MM, Villeneuve R, Tabue Teguo M, Alonso J, Bruffaerts R, Swendsen J, et al. Nursing home workers' mental health during the COVID-19 pandemic in France. J Am Med Dir Assoc. 2022;23(7):1095-100.
31. Khairat S, Zalla LC, Adler-Milstein J, Kistler CE. U.S. nursing home quality ratings associated with COVID-19 cases and deaths. J Am Med Dir Assoc. 2021;22(10):2021-5.e1.
32. Kittang BR, Hofacker SV, Solheim SP, Kruger K, Loland KK, Jansen K. Outbreak of COVID-19 at three nursing homes in Bergen. Tidsskr Nor Laegeforen. 2020;140(11).
33. Kühl A, Hering C, Herrmann WJ, Gangnus A, Kohl R, Steinhagen-Thiessen E, et al. General practitioner care in nursing homes during the first wave of the COVID-19 pandemic in Germany: a retrospective survey among nursing home managers. BMC Prim Care. 2022;23(1):334.
34. Ladhani SN, Chow JY, Janarthanan R, Fok J, Crawley-Boevey E, Vusirikala A, et al. Investigation of SARS-CoV-2 outbreaks in six care homes in London, April 2020. EClinicalMedicine. 2020;26:100533.
35. Levere M, Rowan P, Wysocki A. The adverse effects of the COVID-19 pandemic on nursing home resident well-being. J Am Med Dir Assoc. 2021;22(5):948-54.e2.
36. Li Y, Fang F, He M. Exploring the N95 and surgical mask supply in U.S. nursing homes during COVID-19. J Appl Gerontol. 2021;40(3):257-62.
37. Lipsitz LA, Lujan AM, Dufour A, Abrahams G, Magliozzi H, Herndon L, et al. Stemming the tide of COVID-19 infections in Massachusetts nursing homes. J Am Geriatr Soc. 2020;68(11):2447-53.
38. Louie JK, Scott HM, DuBois A, Sturtz N, Lu W, Stoltey J, et al. Lessons from mass-testing for coronavirus disease 2019 in long-term care facilities for the elderly in San Francisco. Clin Infect Dis. 2021;72(11):2018-20.

39. Marossy A, Rakowicz S, Bhan A, Noon S, Rees A, Virk M, et al. A study of universal severe acute respiratory syndrome coronavirus 2 RNA testing among residents and staff in a large group of care homes in South London. J Infect Dis. 2021;223(3):381-8.
40. Martinchek M, Beiting KJ, Walker J, Graupner J, Huisingh-Scheetz M, Thompson K, et al. Weight loss in COVID-19-positive nursing home residents. J Am Med Dir Assoc. 2021;22(2):257-8.
41. McArthur C, Saari M, Heckman GA, Wellens N, Weir J, Hebert P, et al. Evaluating the effect of COVID-19 pandemic lockdown on long-term care residents' mental health: a data-driven approach in New Brunswick. J Am Med Dir Assoc. 2021;22(1):187-92.
42. McConeghy KW, White E, Panagiotou OA, Santostefano C, Halladay C, Feifer RA, et al. Temperature screening for SARS-CoV-2 in nursing homes: evidence from two national cohorts. J Am Geriatr Soc. 2020;68(12):2716-20.
43. McDermid J, Ballard C, Khan Z, Aarsland D, Fox C, Fossey J, et al. Impact of the Covid-19 pandemic on neuropsychiatric symptoms and antipsychotic prescribing for people with dementia in nursing home settings. Int J Geriatr Psychiatry. 2023;38(1):e5878.
44. McGarry BE, Grabowski DC, Barnett ML. Severe staffing and personal protective equipment shortages faced by nursing homes during the COVID-19 pandemic. Health Aff (Millwood). 2020;39(10):1812-21.
45. Monin JK, Ali T, Syed S, Piechota A, Lepore M, Mourgues C, et al. Family communication in long-term care during a pandemic: lessons for enhancing emotional experiences. Am J Geriatr Psychiatry. 2020;28(12):1299-307.
46. Montoya A, Jenq G, Mills JP, Beal J, Diviney Chun E, Newton D, et al. Partnering with local hospitals and public health to manage COVID-19 outbreaks in nursing homes. J Am Geriatr Soc. 2021;69(1):30-6.
47. Nash WA, Harris LM, Heller KE, Mitchell BD. "We are saving their bodies and destroying their souls.": family caregivers' experiences of formal care setting visitation restrictions during the COVID-19 pandemic. J Aging Soc Policy. 2021;33(4-5):398-413.
48. Navarro Prados AB, Jiménez García-Tizón S, Meléndez JC. Sense of coherence and burnout in nursing home workers during the COVID-19 pandemic in Spain. Health Soc Care Community. 2022;30(1):244-52.
49. Navarro Prados AB, Jiménez García-Tizón S, Meléndez JC, López J. Factors associated with satisfaction and depressed mood among nursing home workers during the covid-19 pandemic. J Clin Nurs. 2024;33(1):265-72.
50. O'Caoimh R, O'Donovan MR, Monahan MP, Dalton O'Connor C, Buckley C, Kilty C, et al. Psychosocial impact of COVID-19 nursing home restrictions on visitors of residents with cognitive impairment: a cross-sectional study as part of the Engaging Remotely in Care (ERiC) Project. Front Psychiatry. 2020;11:585373.
51. Oliveira ACS, Gallego MG, Martínez CG, Martínez EC, Molina JM, Morante JJH, et al. Psychosocial changes during COVID-19 lockdown on nursing home residents, their relatives and clinical staff: a prospective observational study. BMC Geriatr. 2023;23(1):71.
52. Patel MC, Chaisson LH, Borgetti S, Burdsall D, Chugh RK, Hoff CR, et al. Asymptomatic SARS-CoV-2 infection and COVID-19 mortality during an outbreak investigation in a skilled nursing facility. Clin Infect Dis. 2020;71(11):2920-6.
53. Pérez-Rodríguez P, Díaz de Bustamante M, Aparicio Mollá S, Arenas MC, Jiménez-Armero S, Lacosta Esclapez P, et al. Functional, cognitive, and nutritional decline in 435 elderly nursing home residents after the first wave of the COVID-19 pandemic. Eur Geriatr Med. 2021;12(6):1137-45.
54. Pförtner TK, Pfaff H, Hower KI. Will the demands by the Covid-19 pandemic increase the intent to quit the profession of long-term care managers? A repeated cross-sectional study in Germany. J Public Health (Oxf). 2021;43(3):e431-e4.
55. Preuß B, Fischer L, Schmidt A, Seibert K, Hoel V, Domhoff D, et al. COVID-19 in German nursing homes: the impact of facilities' structures on the morbidity and mortality of residents: an analysis of two cross-sectional surveys. Int J Environ Res Public Health. 2022;20(1).
56. Prins M, Willemse B, van der Velden C, Pot AM, van der Roest H. Involvement, worries and loneliness of family caregivers of people with dementia during the COVID-19 visitor ban in long-term care facilities. Geriatr Nurs. 2021;42(6):1474-80.
57. Queiroz BL, Nascimento CQ, Souza TOM, Bádue GS, Bueno NB, Vasconcelos SML, et al. Effects of SARS-CoV-2 infection on health and functional capacity in institutionalized older adults. Revista da Escola de Enfermagem da USP. 2023;57:1-9.

58. Rawle MJ, Bertfield DL, Brill SE. Atypical presentations of COVID-19 in care home residents presenting to secondary care: a UK single centre study. Aging Med (Milton). 2020;3(4):237-44.
59. Riello M, Purgato M, Bove C, MacTaggart D, Rusconi E. Prevalence of post-traumatic symptomatology and anxiety among residential nursing and care home workers following the first COVID-19 outbreak in Northern Italy. R Soc Open Sci. 2020;7(9):200880.
60. Rojo-Perez F, Rodriguez-Rodriguez V, Fernandez-Mayoralas G, Sánchez-González D, Perez de Arenaza Escribano C, Rojo-Abuin JM, et al. Residential environment assessment by older adults in nursing homes during COVID-19 outbreak. Int J Environ Res Public Health. 2022;19(23).
61. Rutten JJS, van Loon AM, van Kooten J, van Buul LW, Joling KJ, Smalbrugge M, et al. Clinical suspicion of COVID-19 in nursing home residents: symptoms and mortality risk factors. J Am Med Dir Assoc. 2020;21(12):1791-7 e1.
62. Ryskina KL, Yun H, Wang H, Chen AT, Jung HY. Characteristics of nursing homes by COVID-19 cases among staff: March to August 2020. J Am Med Dir Assoc. 2021;22(5):960-5 e1.
63. Sacco G, Foucault G, Briere O, Annweiler C. COVID-19 in seniors: findings and lessons from mass screening in a nursing home. Maturitas. 2020;141:46-52.
64. Savci C, Cil Akinci A, Yildirim Usenmez S, Keles F. The effects of fear of COVID-19, loneliness, and resilience on the quality of life in older adults living in a nursing home. Geriatr Nurs. 2021;42(6):1422-8.
65. Schulze S, Merz S, Thier A, Tallarek M, König F, Uhlenbrock G, et al. Psychosocial burden in nurses working in nursing homes during the Covid-19 pandemic: a cross-sectional study with quantitative and qualitative data. BMC Health Serv Res. 2022;22(1):949.
66. Senczyszyn A, Lion KM, Szczesniak D, Trypka E, Mazurek J, Ciulkowicz M, et al. Mental health impact of SARS-COV-2 pandemic on long-term care facility personnel in Poland. J Am Med Dir Assoc. 2020;21(11):1576-7.
67. Shi SM, Bakaev I, Chen H, Travison TG, Berry SD. Risk factors, presentation, and course of coronavirus disease 2019 in a large, academic long-term care facility. J Am Med Dir Assoc. 2020;21(10):1378-83 e1.
68. Takahashi T, Ekoyama S, Tachikawa H, Midorikawa H, Shiratori Y, Ota M, et al. Mental health of caregivers working in nursing homes during the COVID-19 pandemic. Dement Geriatr Cogn Disord. 2022;51(3):233-40.
69. Tang O, Bigelow BF, Sheikh F, Peters M, Zenilman JM, Bennett R, et al. Outcomes of nursing home COVID-19 patients by initial symptoms and comorbidity: results of universal testing of 1970 residents. J Am Med Dir Assoc. 2020;21(12):1767-73 e1.
70. Tebbeb N, Villemagne F, Prieur T, Dorier S, Fort E, Célarier T, et al. COVID-19 health crisis workloads and screening for psychological impact in nursing home staff: a qualitative and quantitative survey. Int J Environ Res Public Health. 2022;19(7).
71. Telford CT, Bystrom C, Fox T, Holland DP, Wiggins-Benn S, Mandani A, et al. COVID-19 infection prevention and control adherence in long-term care facilities, Atlanta, Georgia. J Am Geriatr Soc. 2021;69(3):581-6.
72. Urlings JHJ, Backhaus R, Verbeek H, de Boer B, Koopmans R, Gerritsen DL, et al. After COVID-19 vaccinations: what does living and working in nursing homes look like? BMC Geriatr. 2023;23(1):298.
73. van der Roest HG, Prins M, van der Velden C, Steinmetz S, Stolte E, van Tilburg TG, et al. The impact of COVID-19 measures on well-being of older long-term care facility residents in the Netherlands. J Am Med Dir Assoc. 2020;21(11):1569-70.
74. van Dijk Y, Janus SIM, de Boer MR, Achterberg WP, Roelen CAM, Zuidema SU. Job demands, work functioning and mental health in Dutch nursing home staff during the COVID-19 outbreak: a cross-sectional multilevel study. Int J Environ Res Public Health. 2022;19(7).
75. Vandael E, Latour K, Islamaj E, Panis LI, Callies M, Haarhuis F, et al. COVID-19 cases, hospitalizations and deaths in Belgian nursing homes: results of a surveillance conducted between April and December 2020. Archives of Public Health. 2022;80(1):1-12.
76. Verbeek H, Gerritsen DL, Backhaus R, de Boer BS, Koopmans R, Hamers JPH. Allowing visitors back in the nursing home during the COVID-19 crisis: a Dutch national study into first experiences and impact on well-being. J Am Med Dir Assoc. 2020;21(7):900-4.
77. von der Warth R, Brühmann BA, Farin-Glattacker E. The association of care satisfaction and COVID-19 contact restrictions with quality of life in long-term care homes residents in Germany: a cross-sectional study. Eur Geriatr Med. 2022;13(6):1335-42.

78. Wammes JD, Kolk D, van den Besselaar JH, MacNeil-Vroomen JL, Buurman-van Es BM, van Rijn M. Evaluating perspectives of relatives of nursing home residents on the nursing home visiting restrictions during the COVID-19 crisis: a Dutch cross-sectional survey study. J Am Med Dir Assoc. 2020;21(12):1746-50.e3.
79. Watts T, Holston EC, Yimmee S. Quality of life health outcomes among people who lived in a nursing home prior to and during the COVID-19 pandemic. J Gerontol Nurs. 2023;49(12):41-8.
80. Xu H, Intrator O, Bowblis JR. Shortages of staff in nursing homes during the COVID-19 pandemic: what are the driving factors? J Am Med Dir Assoc. 2020;21(10):1371-7.
81. Yan D, Temkin-Greener H, Cai S. Did the COVID-19 pandemic affect the use of antipsychotics among nursing home residents with ADRD? Am J Geriatr Psychiatry. 2023;31(2):124-40.
82. Ye P, Fry L, Champion JD. Changes in advance care planning for nursing home residents during the COVID-19 pandemic. J Am Med Dir Assoc. 2021;22(1):209-14.
